# A low carbohydrate diet high in fish oil and soy protein delays inflammation, hematopoietic stem cell depletion, and mortality in miR-146a knock-out mice

**DOI:** 10.3389/fnut.2022.1017347

**Published:** 2022-11-24

**Authors:** Ingrid Elisia, Sara Kowalski, Michelle Yeung, Jennifer Wong, Jennifer M. Grants, Aly Karsan, Gerald Krystal

**Affiliations:** ^1^The Terry Fox Laboratory, BC Cancer Research Centre, Vancouver, BC, Canada; ^2^Canada’s Michael Smith Genome Sciences Centre, BC Cancer Research Centre, Vancouver, BC, Canada

**Keywords:** miR-146a, low carbohydrate diet, inflammation, HSC exhaustion, stem cell

## Abstract

Since our previous studies found a low carbohydrate (CHO) diet containing soy protein and fish oil (15%Amylose/Soy/FO) significantly reduced lung and breast cancer in mice we asked herein if this low CHO diet could also delay the onset of myeloid malignancies. To test this we employed a miR-146a knock-out (KO) mouse model and found the 15%Amylose/Soy/FO diet increased their median lifespan by 8.5 month, compared to these mice on a Western diet. This was associated with increased lymphocytes and reduced monocytes, granulocytes, blood glucose and insulin levels. Inflammatory cytokine/chemokine studies carried out with 6-month-old mice, before any signs of illness, revealed the 15%Amylose/Soy/FO diet significantly reduced pro-inflammatory cytokines. This low CHO diet also led to an increase in plasma β-hydroxybutyrate and in liver fatty acid synthase levels. This, together with higher liver carnitine palmitoyltransferase I levels suggested that the 15%Amylose/Soy/FO diet was causing a systemic metabolic shift from glucose to fatty acids as an energy source. Lastly, we found the 15%Amylose/Soy/FO diet resulted in significantly higher numbers of primitive hematopoietic stem cells (HSCs) in the bone marrow of 6-month-old mice than those fed a Western diet. Taken together, these results suggest a 15%Amylose/Soy/FO diet reduces chronic inflammation and increases fatty acid oxidation and that this, in turn, may prevent HSC proliferation and exhaustion, thereby delaying myeloid malignancy-induced death of miR-146a KO mice. We suggest a low CHO diet containing soy protein and fish oil could be beneficial in reducing the risk of myeloid malignancies in patients with low miR-146a levels.

## Introduction

Since cancer cells typically consume more glucose than normal cells to meet their energy requirements and to generate cellular building blocks for proliferation (Warburg Effect), we hypothesized, many years ago, that low carbohydrate (CHO) diets might both slow the growth of established tumors and prevent the survival of nascent cancer cells. Our early studies confirmed that diets low in CHO were indeed effective in both slowing the growth of subcutaneously implanted tumors in mice and in preventing the appearance of tumors in mice genetically predisposed to develop various cancers (1, 2). To determine if the effect of a low CHO diet was influenced by the type and level of macronutrients present, we also compared diets differing in both the level and type of CHO, protein and fat (2). These diets were evaluated using a tobacco carcinogen (nicotine-derived nitrosamine ketone, NNK) induced lung cancer model in A/J mice. We found that a diet composed of 15% (of total calories) as CHO, containing primarily the resistant starch, amylose, 35% soy protein and 50% fat, with the majority being omega 3-rich fish oil (i.e., 15%Amylose/Soy/FO) was the most effective at both preventing lung nodule formation and slowing the growth of established lung nodules (2, 3). When compared to a Western diet, this 15%Amylose/Soy/FO chow was also effective in slowing breast cancer growth in female C3(1)/Tag mice, a transgenic mouse strain that spontaneously develops mammary tumors resembling triple-negative breast cancer (4). In addition to being able to lower blood glucose and insulin in both these mouse models, this 15%Amylose/Soy/FO diet also reduced plasma levels of IL-6, TNFα and PGE_2_ levels (3, 4), suggesting it possessed anti-inflammatory properties.

To broaden the scope of our studies we asked if this low CHO diet could also prevent or slow the development of other cancers. With this in mind we investigated, herein, whether this 15%Amylose/Soy/FO diet could influence myeloid malignancies. Specifically, we compared the effect of a Western diet to the 15%Amylose/Soy/FO diet on the survival of the miR-146a KO mice, a mouse model which succumbs to myeloid malignancies frequently associated with myelodysplastic syndromes (MDS). Myelodysplastic syndromes (MDS) are a heterogeneous group of blood cancers that arise due to mutations in hematopoietic stem cells (HSCs) that hinder their differentiation to normal mature blood cells in the bone marrow (5). MDS patients typically experience bone marrow failure or progress to acute myeloid leukemia (AML) (5). For the studies presented herein we used the miR-146a knock-out (KO) mouse model (6). MiR-146a, located on human chromosome 5q, is implicated in the pathogenesis of MDS, particularly the del(5q) MDS subtype (7, 8). It is a potent negative regulator of inflammation *via* repression of TRAF6, and IRAK1, two upstream positive regulators of NF-κB, a master transcription factor that stimulates the expression of pro-inflammatory cytokines (9). Interestingly, NF-κB also induces miR-146a, suggesting a negative feedback loop to ensure pro-inflammatory cytokine production is appropriately turned off after an infection is eliminated (9). Of interest clinically, reduced expression of miR-146a in AML patients’ bone marrow is associated with increased inflammatory signaling and poor outcome (10). In keeping with these clinical findings, miR-146a KO mice have been shown to develop increased levels of pro-inflammatory cytokines such as IL-6 and TNFα and develop a myeloid malignancy that significantly reduces their lifespan (6, 10, 11). There are also intrinsic changes within the HSCs of miR-146a mice, making then more sensitive than wild type (WT) mice to IL-6-induced cycling (10). Importantly, reducing inflammation in miR-146a KO mice, by concomitant deletion of *Tnf* or *Il6*, has been shown to extend their lifespan by delaying the onset of myeloid malignancy (10).

Since chronic inflammation has been shown to contribute to the development of myeloid malignancies (12), we hypothesized that a 15%Amylose/Soy/FO diet might ameliorate chronic inflammation in miR-146a KO mice and thus both delay the development of myeloid malignancy and extend the lifespan of these mice.

## Materials and methods

### Animals

Female miR-146a KO or female syngeneic wild type (WT) C57BL/6 mice were weaned at 3 weeks of age onto a standard Envigo #2920 chow and then transferred onto a Western or 15%Amylose/Soy/FO chow at 5 weeks of age. The ingredients of these two diets are shown in Table 1 and Supplementary Tables 1–4. Ten to fifteen mice/cage were housed in double-decker cages consisting of two rat cages stacked on top of each other with a hole between them for access and an exercise wheel on each floor. The food and water bottle were in the food hopper of the top cage and the mice were allowed to consume their diets *ad libitum*. All animal experiments were approved by the University of British Columbia Animal Care Committee (A18-0138).

**TABLE 1:** Diet formulation expressed as g/kg of diet.

	Western	15% Amylose, Soy, FO
**Carbohydrate**		
Corn starch	230	0
High amylose corn starch	0	100
Maltodextrin	70	70
Sucrose	255	0
Cellulose	30	153
**Protein**		
Casein	190	0
Soy protein isolate	0	396
**Fat**		
Soybean oil	31.4	42.1
Milk fat	36.3	12.1
Olive oil	28	9.3
Lard	28	9.3
Beef tallow	24.8	8.3
Corn oil	16.5	5.5
Fish oil	0	134.5
**Kcal/g**	4.4	4
Carbohydrate (kcal %)	50	15.4
Protein (kcal %)	15.4	34.5
Fat (kcal %)	34.5	50.4

### Study 1. Survival of miR-146a knock-out mice on different diets

The mice were maintained on their respective diets and weighed once/week until they reached their humane endpoint, which, for these miR-146a KO mice, was typically when they became moribund and immobile with hind leg paralysis/weakness. At this time, the mice were euthanized by CO_2_ asphyxiation while under isoflurane, followed by cervical dislocation. The mice were subsequently cardiac-punctured and blood, collected into EDTA tubes, was centrifuged at 2,500 × g for 10 min at 4°C and the plasma was stored at −80°C until further analysis. The organs (liver, kidney and spleen) were weighed and frozen at −80°C. Bone marrow from the femurs and tibias were flushed with PBS containing 2% FCS and the red blood cells were lysed *via* incubation for 5 min with a cold ammonium chloride solution (StemCell Technologies, Vancouver, Canada) in a 2:1 ratio. After washing with PBS containing 2% FCS, the white blood cells were counted, centrifuged at 2,500 × g for 5 min at 4°C and the pellets frozen at −80°C.

### Study 2. The effect of diets on systemic inflammation and ESLAM levels

To determine the effect of the diets on systemic inflammation and the levels of HSCs (i.e., “ESLAM” cells, described below) prior to the onset of myeloid malignancy, we repeated Study 1 but euthanized the mice when they reached 6 months of age rather than at their humane endpoint. We chose 6 months because Study 1 suggested that these mice did not show any signs of morbidity until at least 9 months of age. We euthanized these mice as described above, obtained plasma from cardiac-punctured blood, and weighed organs. The bone marrow cells were also obtained as described above, but then enriched for HSCs by lineage depletion using an EasySep™ mouse hematopoietic progenitor cell isolation kit (#19856, StemCell Technologies, Vancouver, Canada). The cells were subsequently stained for surface markers to quantify the CD45^+^EPCR^+^CD150^+^CD48^–^ (ESLAM) population using flow cytometry as previously described (10).

### EPCR-high/CD150-high ESLAM subpopulation analysis

Within the ESLAM population, a subpopulation with higher expression of the ESLAM markers EPCR and CD150 has been shown to have enhanced HSC stemness and be depleted in miR-146a KO relative to WT mice (10). To identify if the diets impacted this specific ESLAM subpopulation, ESLAMs were gated based on fluorescence minus one (FMO) controls using FlowJo software, and exported for further analysis in RStudio. Samples with low numbers of ESLAMs (≤125 events; *n* = 4 miR-146a KO Western diet samples) were excluded from further analysis. The standard deviation of the median EPCR-PE (822 ± 8.6) and CD150-PE/Cy7 (708 ± 10.6) values was low across all experimental replicates; therefore, normalization across replicates was not performed. EPCR-high/CD150-high ESLAMs were defined as ESLAMs with EPCR-PE and CD150-PE/Cy7 values greater than the median values observed in ESLAMs from WT animals. The percentage of EPCR-high/CD150-high ESLAMs was calculated per animal.

### Blood cell differential analysis

Once the mice reached 6 months of age a complete blood count was performed once a month until the mice reached their humane endpoints blood the tail-vein using a scil Vet hematology analyzer (Viernheim, Germany).

### Measurement of blood glucose, inflammatory markers, and other biochemical analysis

Blood glucose levels were measured from the tail vein-collected blood as previously described, when mice were actively feeding between 8 p.m. and 11 p.m. (2). Beta-hydroxybutyrate (β-HB), insulin, and PGE2 levels in mouse plasma from cardiac punctured blood in Study 1 and saphenous blood in Study 2, were measured using ELISA kits as previously described (2). The levels of the pro-inflammatory cytokines/chemokines MIP1α, MIP2, IL-15, IFNγ, IL-1β, IL-2, IL-4, IL-5, IL-6, KC/Gro, IL-10, IL-12p70, and TNFα in the plasma were quantified using Mesoscale V-plex pro-inflammatory kits (K152AOH-1 and K15048D-2).

### Western blotting of liver FAS and CPT1a

Livers of 6-month-old mice on Western and 15%Amylose/Soy/FO diets were subjected to SDS-PAGE and Western blotting as described previously (2).

### Statistics

To identify significant differences in end-point measures between mice fed the Western vs. the 15%Amylose/Soy/FO chow, we performed a two-tailed unpaired *t*-test. *P*-values lower than 0.05 were considered statistically significant. The effect of the two diets on the survival of the mice was plotted using the Log-rank (Mantel-Cox) test. Statistical analysis was performed using GraphPad Prism 9.2 (GraphPad Software, Inc.).

## Results

### A 15%Amylose/Soy/FO diet extends the lifespan of miR-146a knock-out mice

**FIGURE 1:**
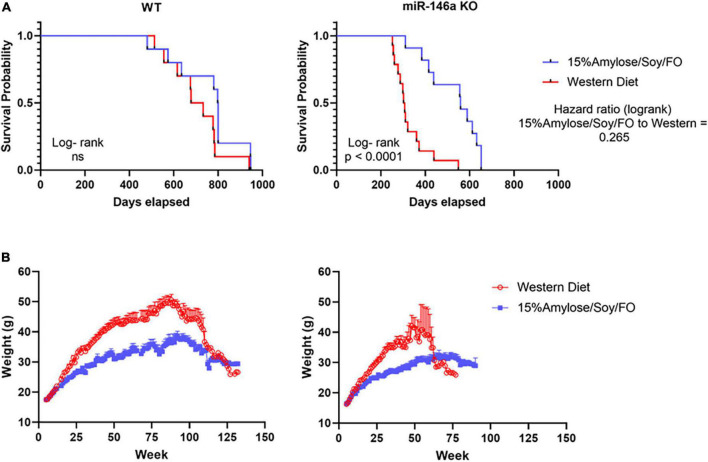
A 15%Amylose/Soy/FO diet extends the lifespan and reduces the weight of miR-146a KO mice. At 5 weeks of age miR-146a KO (left panel) or WT (right panel) C57Bl/6 mice were housed in two-tiered rat cages with exercise wheels and put on a Western (15 mice) or the 15%Amylose/Soy/FO diet (15 mice) and their **(A)** survival monitored and **(B)** their weights monitored weekly.

We first compared the lifespan of these KO mice fed Western vs. 15%Amylose/Soy/FO chow. As shown in the left panel of Figure 1A, miR-146a KO mice fed the 15%Amylose/Soy/FO diet had a median survival of 559 days while those fed the Western diet had a median survival of only 304 days. On the other hand, when we compared the effect of these two diets on the lifespan of syngeneic WT C57BL/6 mice we did not find a significant difference, although there was a trend (*p* < 0.07) toward a longer lifespan with the low CHO diet (799 days for the 15%Amylose/Soy/FO diet vs. 705 days for the Western diet) (Figure 1A). Also worthy of note is that while the 15%Amylose/Soy/FO diet lengthened the lifespan of the miR-146a KO mice it did not bring it back to that seen in WT mice Figure 1A).

To gain some insight into why the miR-146a KO mice on a 15%Amylose/Soy/FO diet were living longer we first examined their body weights over time. As shown in Figure 1B, the miR-146a KO mice on a Western diet became significantly heavier than those on the 15%Amylose/Soy/FO diet, starting at about 10 weeks of age. A similar difference in weight was also seen with WT mice, suggesting that the deletion of miR-146a did not play a role in this diet-induced weight difference.

### A 15%Amylose/Soy/FO diet ameliorates the blood cell profile changes associated with miR-146a knock-out mice

**FIGURE 2:**
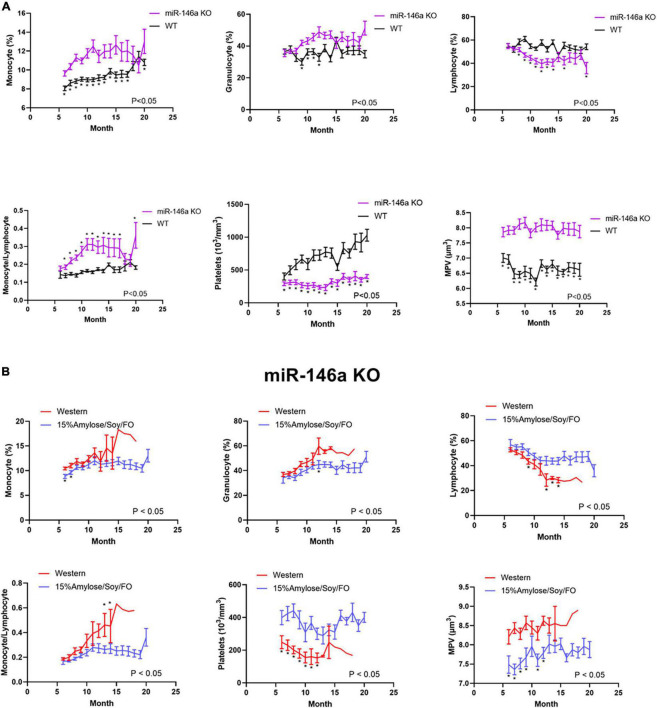
A 15%Amylose/Soy/FO diet ameliorates the blood cell profile changes associated with miR-146a KO mice. **(A)** A comparison of the lymphocytes, monocytes, granulocytes, and platelets over time in WT and miR-146a KO mice on a Western diet. The data are shown as the means ± SEM of 10 WT and 15 miR-146a KO mice. **(B)** A comparison of the lymphocytes, monocytes, granulocytes and platelets over time in miR-146a KO mice on a Western (*n* = 15) vs. a 15%Amylose/Soy/FO (*n* = 15) diet. The data are shown as the means ± SEM. *Indicates significant differences (*P* < 0.05).

To determine if the 15%Amylose/Soy/FO diet brought blood cell profiles in miR-146a KO mice closer to that in WT mice, we first compared WT vs. miR-146a KO mouse blood cells profiles, over time, with both mouse types on Western diets. As shown in Figure 2A, when mice were followed from 6 months of age to their humane endpoint, the miR-146a KO mice had significantly (*P* < 0.05) lower lymphocytes and higher monocytes and granulocytes than WT mice, in keeping with increased inflammation. Also of note, the WT mice on a Western diet increased their platelets with age, starting at 6 months of age, while the miR-146a KO mice on the same diet retained low platelet levels over time (13).

We next compared the blood cell profiles of the miR-146a KO mice fed the 15%Amylose/Soy/FO vs. Western diet over time and observed marked differences. Specifically, as shown in Figure 2B, Mice fed the 15%Amylose/Soy/FO chow had significantly (*P* < 0.05) higher lymphocyte and lower monocyte and granulocyte proportions, as well as higher platelet counts and lower MPVs than Western-fed miR-146a KO mice, bringing their blood cell profile closer to that present in WT mice (Supplementary Figure 1). The effect of the two diets on WT mice was not nearly as pronounced and, in fact, only minor differences in blood cell profiles were detected (Supplementary Figure 2). This indicates that it is the deletion of miR-146a that is responsible for the lower platelet numbers, the increased MPV and the higher granulocyte and monocyte/lymphocyte ratio. Moreover, these results suggest that the 15%Amylose/Soy/FO diet brings these blood parameters in miR-146a KO mice closer to that present in WT mice. Thus, this effect on blood profiles might play a role in the extension of lifespan seen in miR-146a KO mice on the 15%Amylose/Soy/FO diet.

### A 15%Amylose/Soy/FO diet reduces weight gain, spleen enlargement, blood glucose, and insulin levels, while increasing β-hydroxybutyrate in 6-month-old miR-146a knock-out mice

**FIGURE 3:**
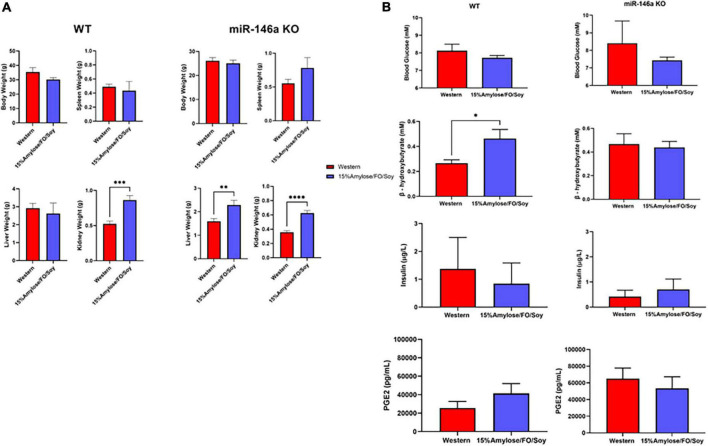
A 15%Amylose/Soy/FO diet reveals only minor differences with a Western diet when miR-146a KO or WT mice are compared at their humane endpoints. **(A)** A comparison of the body, spleen, liver and kidney weights of both WT mice on a Western (*n* = 10) vs. a 15%Amylose/Soy/FO (*n* = 10) diet and miR-146a KO mice on a Western (*n* = 15) vs. a 15%Amylose/Soy/FO (*n* = 15) diet when the mice reached their humane endpoint. **(B)** Blood glucose, insulin, β-HB and PGE_2_ levels of both WT mice on a Western (*n* = 10) vs. a 15%Amylose/Soy/FO (*n* = 10) diet and miR-146a KO mice on a Western (*n* = 15) vs. a 15%Amylose/Soy/FO (*n* = 15) diet when the mice were within 1 month of their humane endpoint. The data are shown as the mean ± SEM. *P < 0.05, **P < 0.01, ****P < 0.0001.

When WT and miR-146a KO mice reached their humane endpoints and were euthanized, the livers, kidneys and spleens were weighed and, as shown in Figure 3A, kidneys were significantly heavier on the 15%Amylose/Soy/FO diet in both WT and miR-146a KO mice (Table 1) (1). The enlarged kidneys were likely a benign response to the high protein content in the 15%Amylose/Soy/FO (14), since a similar increase in kidney weight was observed in earlier studies with different mouse strains fed this same low CHO high protein diet (2, 4) and this was not associated with kidney damage [i.e., albumin secretion (1)].

**FIGURE 4:**
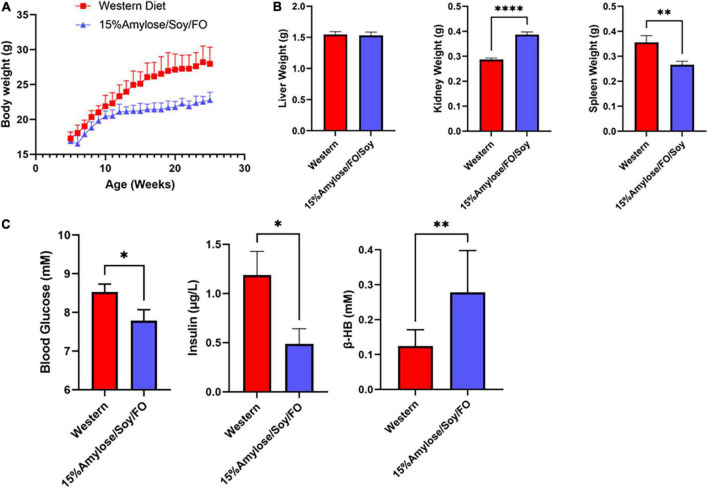
A 15%Amylose/Soy/FO diet reduces body weight, spleen enlargement, blood glucose, and insulin levels and increases β-hydroxybutyrate in 6-month-old miR-146a KO mice. **(A)** At 5 weeks of age, miR-146a KO mice were housed in two-tiered rat cages with exercise wheels and put on a Western (10 mice) or the 15%Amylose/Soy/FO diet (10 mice) and their weights monitored weekly. **(B)** Liver, kidneys and spleens were weighed following euthanasia of the miR-146a KO mice on the two diets at 6 months of age. **(C)** Blood glucose, insulin, and β-HB levels were monitored 1 week prior to euthanasia. The data are shown as the mean +/– SEM. *P < 0.05, **P < 0.01, ****P < 0.0001.

An examination of blood glucose, insulin, β-hydroxybutyrate (β-HB) and PGE_2_ levels (Figure 3B) yielded results that were inconsistent with our previous studies comparing Western and 15%Amylose/Soy/FO in which we found significantly lower blood glucose and insulin levels on the 15%Amylose/Soy/FO diet (2, 4). We therefore questioned whether comparing these parameters at the humane endpoints of these mice, when they were ill, was appropriate. We therefore repeated this study with miR-146a KO mice on either a Western or 15%Amylose/Soy/FO diet and sacrificed them this time at 6 months of age, before any significant changes in blood cell profiles or morbidity appeared to confound our results. As shown in Figure 4, miR-146a KO mice once again gained significantly more weight over time on the Western diet. However, spleen weights in the miR-146a KO mice at 6 months of age, this time, were significantly greater on the Western compared to the 15%Amylose/Soy/FO diet (Figure 4). We also evaluated blood glucose, insulin and β-HB levels of miR-146a KO mice on the two diets (Figure 4C). As expected, given the higher level of easily digestible CHO in the Western diet, there were significantly higher blood glucose levels on this diet. Consistent with this, there were also significantly higher insulin levels in the Western diet-fed mice. As well, there was a significant increase in β-HB levels in the miR-146a KO mice on the 15%Amylose/Soy/FO diet, suggesting a systemic switch from glucose to fatty acids and ketone bodies to meet their metabolic needs (15).

### A 15%Amylose/Soy/FO diet reduces inflammatory cytokines in 6-month-old miR-146a knock-out mice

**FIGURE 5:**
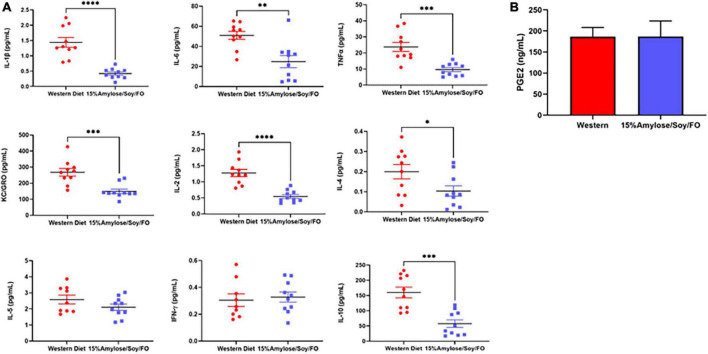
A 15%Amylose/Soy/FO diet reduces inflammatory cytokines in 6-month-old miR-146a KO mice. **(A)** Mesoscale analysis of IL-1β, IL-2, IL-4, IL-5, IL-6, KC/Gro, TNFα, IFNγ, and IL-10 plasma levels from miR-146a KO mice on a Western vs. a 15%Amylose/Soy/FO diet at 6 months of age. The data are shown as the mean ± SEM of 10 miR146a KO mice on a Western and 10 miR146a KO mice on a 15%Amylose/Soy/FO diet. **(B)** Plasma PGE_2_ levels of miR146a KO mice on a Western vs. a 15%Amylose/Soy/FO diet at 6 months of age. The data are shown as the mean ± SEM of 10 miR146a KO mice on a Western and 10 miR146a KO mice on a 15%Amylose/Soy/FO diet. NS = not significant, *P < 0.05, **P < 0.01, ***P < 0.001, ****P < 0.0001.

Since miR-146a has been shown to be an important anti-inflammatory microRNA (9, 16, 17) and its deletion drives a pro-inflammatory state that is thought to lead to MDS (8, 10, 18, 19), we compared the effect of the two diets on a number of pro-inflammatory cytokines/chemokines in the plasma from 6-month-old miR-146a KO mice. As shown in Figure 5A, mice fed 15%Amylose/Soy/FO had significantly (*P* < 0.05) lower levels of the pro-inflammatory cytokines IL-6, TNFα, IL-1β, and the chemokine KC/Gro (aka CXCL1, the mouse homolog of human IL-8) than mice fed the Western diet. Also, while there was no significant difference in IFNγ levels, the 15%Amlose/Soy/FO fed mice also had significantly (*P* < 0.05) lower IL-2, IL-4 and IL-10 levels. Of note, we also measured plasma MCP-1 levels from mice euthanized at 6 months old and found that the levels of this chemokine were not significantly impacted by the diet. The higher level of the anti-inflammatory cytokine, IL-10, on the Western diet is particularly interesting and may represent an attempt to counter the elevated pro-inflammatory cytokines on the Western diet (20). These results are in marked contrast to cytokine/chemokine levels of miR-146a KO mice taken at their humane endpoints (Supplementary Figure 3), where we saw no significant differences in IL-6, TNFα, IL-1β, IL-2, IL-4, IL-5 or IL-10 between mice on the two diets. Only KC/Gro was consistently reduced on a 15%Amylose/Soy/FO diet at both 6 months and their humane endpoint (Supplementary Figure 3 and Figure 5). This supports examining inflammatory markers before the appearance of any signs of morbidity.

We did not find PGE_2_ levels in the plasma of 6-month-old mice to be impacted by the diets (Figure 5B), suggesting that the efficacy of the 15%Amylose/Soy/FO diet at extending the lifespan of miR-146a KO mice is likely not attributable to its effect on the COX2 signaling pathway (2, 4).

### A 15%Amylose/Soy/FO diet reduces liver fatty acid synthase and increases carnitine palmitoyltransferase I in 6-month-old miR-146a knock-out mice

**FIGURE 6:**
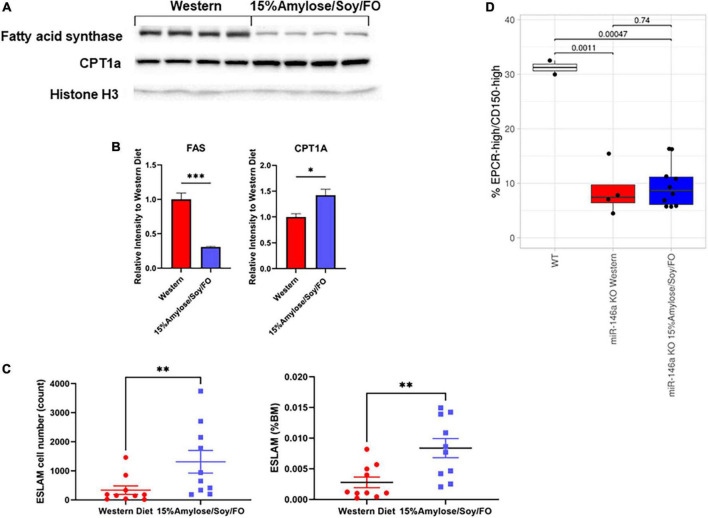
A 15%Amylose/Soy/FO diet skews energy production to fatty acid/ketone body hydrolysis and increases the number of hematopoietic stem cells in 6-month-old miR-146a KO mice. **(A)** A Western blot showing FAS and CPT1 protein levels in the livers from 4 mice on a Western vs. 4 mice on a 15%Amylose/Soy/FO diet, taken at 6 months of age. Histone H3 was used as a loading control. **(B)** Densitometry plots of the FAS and CPT1 levels divided by the histone H3 levels. *P < 0.05, **P < 0.01, ***P < 0.001. **(C)** Flow cytometric analysis of primitive HSCs, based on EPCR+CD45+CD150+CD48- (ESLAM) markers, within the bone marrow of 6-month-old miR-146a KO mice on a Western vs. a 15%Amylose/Soy/FO diet. The data are shown as the mean ± the SEM from 8 mice on a Western and 10 mice on a 15%Amylose/Soy/FO diet and expressed as absolute numbers of HSCs per mouse (left panel) or percent of total nucleated bone marrow cells (right panel). **(D)** The level of EPCR^hi^/CD150^hi^ ESLAMs as a percent of total ESLAMs from mice with ESLAMs ≥ 125 events. *P*-values from *t*-test analysis were expressed between the groups being compared.

Our finding that the 15%Amylose/Soy/FO diet led to a significant increase in plasma β-HB levels in the miR-146a KO mice (Figure 4C) suggested a shift in these mice from a dependency on glucose as an energy source to fatty acids and ketone bodies. To explore this further we carried out Western blots of liver samples from miR-146a KO mice and found that the 15%Amylose/Soy/FO diet significantly reduced the protein level of FAS and increased that of CPT1a (Figures 6A,B). This is of interest because the enzyme, FAS, facilitates fat synthesis and storage when glucose is abundant, while CPT1a does the opposite, promoting fatty acid β-oxidation and breakdown for energy (15, 21, 22). Thus, these results are consistent with our β-HB findings and strengthen the hypothesis that miR-146a KO mice on a 15%Amylose/Soy/FO diet shift from using glucose to using fatty acids and ketone bodies as an energy source.

### A 15%Amylose/Soy/FO diet increases the number of hematopoietic stem cells in 6-month-old miR-146a knock-out mice

Since we observed significantly different blood cell profiles in the miR-146a KO mice on the two diets (11), we hypothesized that these diets might also affect hematopoiesis at the stem cell level. To test this, we quantified the frequency of primitive HSCs, based on the ESLAM immunophenotype, in 6-month-old miR-146a KO mice on the two diets. As shown in Figure 6C, the bone marrow of miR-146a KO mice on the 15%Amylose/Soy/FO diet had both a significantly (*P* < 0.05) higher absolute number (1,313 ± 389, mean ± SEM) than on the Western diet (339 ± 146) as well as a higher percent of ESLAM cells. Similar aged WT mice fed standard chow had 1,401 ± 89 ESLAM cells. Despite the significant difference in the total ESLAM number in mice fed with the two diets, further characterization of these ESLAM cells showed that the proportion of the highly quiescent (EPCR*^hi^*/CD150*^hi^*)(10) subpopulation was comparable (Figure 6D), suggesting no selective retention of high or low quiescent HSC subpopulations by this low carbohydrate diet. Based on our finding that mice fed the 15%Amylose/Soy/FO diet also had a significantly lower level of circulating pro-inflammatory cytokines and lower monocyte percentages in the blood than mice on the Western diet, this is consistent with the 15%Amylose/Soy/FO diet lowering both chronic inflammation-induced proliferation/differentiation and subsequent exhaustion of HSCs.

## Discussion

Based on evidence that chronic inflammation plays an important role in the development of MDS (23) and our earlier studies showing a 15%Amylose/Soy/FO diet reduces chronic inflammation in various mouse cancer models (2, 4), we hypothesized that this low CHO diet might delay the development of myeloid malignancy and thus extend the lifespan of miR-146a KO mice. Consistent with this hypothesis we found that the median survival of miR-146a KO mice on a 15%Amylose/Soy/FO diet was 8.5 months longer than these same mice on a Western diet, reducing the risk of death by 74% (i.e., a hazard ratio of 15%Amylose/Soy/FO to Western = 0.265). As well, a comparison of blood cell profiles over time revealed that a 15%Amylose/Soy/FO diet brought the increased numbers of monocytes and granulocytes and reduced numbers of lymphocytes and platelets seen with miR-146a KO mice on a Western diet closer to that present in WT mice. This fits with reports showing that chronic inflammation induces a myeloid bias of HSPCs (24).

It should be noted, however, that the blood cell profiles of the miR-146a KO mice fed the Western or the 15%Amylose/Soy/FO diet were not significantly different at their humane endpoints, and they exhibited a comparable degree of splenomegaly. We also observed, just before these miR-146a KO mice required euthanasia, regardless of their diet, a precipitous drop in platelets and hind-leg paralysis, suggesting that the 15%Amylose/Soy/FO primarily delayed myeloid-malignancy-induced death rather than change the nature of the malignancy. Exploring why the 15%Amylose/Soy/FO diet was extending the lifespan of miR-146a KO mice, we found a significant weight gain on the Western diet (2), which is important because obesity in itself, *via* secretion of inflammatory cytokines from macrophages in visceral fat, increases chronic inflammation (25–27).

When plasma cytokines/chemokines were measured at the humane endpoints of the miR-146a KO mice, we obtained negligible differences between the two diets. This suggested that these levels may not be representative of the levels present before any significant changes in blood cell profiles and overall health appeared. For this reason, we repeated the study to measure the impact of the two diets on the inflammation status of the miR-146a KO mice before major differences in blood cell profiles, other than in platelets, were detected, i.e., at 6 months of age. Results from this second study revealed that the 15%Amylose/Soy/FO diet significantly reduced IL-6, TNFα and IL-1β, as well as other pro-inflammatory cytokines and chemokines, compared to a Western diet. Of note, however, even the elevated plasma cytokines of the mice on the Western diet at 6 months of age (Figure 5A) were significantly below those present at the humane endpoints of these mice (Supplementary Figure 3). This is important because IL-6, TNFα and IL-1β are the primary cytokines associated with the increase in chronic inflammation that occurs with age, i.e., “inflammaging” and with systemic age-related functional decline and mortality in multiple studies (28–33). Taken together, these results suggest that the 15%Amylose/Soy/FO diet delays inflammaging. Related to this, Grants et al showed that targeting IL-6 or TNFα, or their upstream regulator, NF-kB, restored HSC function and reduced the development of hematological malignancies in miR-146a KO mice (10).

Our finding that primitive HSCs (i.e., ESLAMs) were present at significantly higher numbers in the bone marrow of 6 month-old miR-146a KO mice on a 15%Amylose/Soy/FO than a Western diet is consistent with previous studies showing that the chronic inflammation present in miR-146a KO mice is associated with premature HSC aging (10). In this regard, Zhao et al. (11, 19) have shown that during inflammation, caused either by infections or inflammaging, HSCs and progenitor cells are stimulated to proliferate and differentiate into mature immune cells, especially of the myeloid lineage. This is consistent with the elevations in monocytes and granulocytes we observed with miR-146a KO mice on a Western diet and the amelioration of this increase in mature myeloid cells with the 15%Amylose/Soy/FO diet. Zhao et al. (11) found that the increased inflammation in miR-146a KO mice leads to a transient increase in HSCs (at 4 months of age) but, by 8 months this results in a significant decrease in HSCs which becomes progressively more severe and by 12 months of age there is only a residual number of CD45+ bone marrow cells and nearly complete exhaustion of HSCs (11). Importantly, these HSCs have an increased sensitivity to pro-inflammatory cytokines, accelerating their proliferation, differentiation and exhaustion (10). Our results suggest that the 15%Amylose/Soy/FO diet, by reducing pro-inflammatory cytokines, likely delays the exhaustion of both highly quiescent (EPCR^hi^/CD150^hi^) and less quiescent ESLAMs to the same extent.

Our finding that the 15%Amylose/Soy/FO diet appears to be shifting systemic metabolism from glycolysis to FAO is relevant to the fate of HSCs since it has been shown that FAO promotes normal HSC self-renewal (34). FAO also appears beneficial for HSCs that typically rely on glycolysis in their quiescent state, since it generates NADPH that can be used to protect against ROS and thereby prevent proliferation/differentiation (35).

**FIGURE 7:**
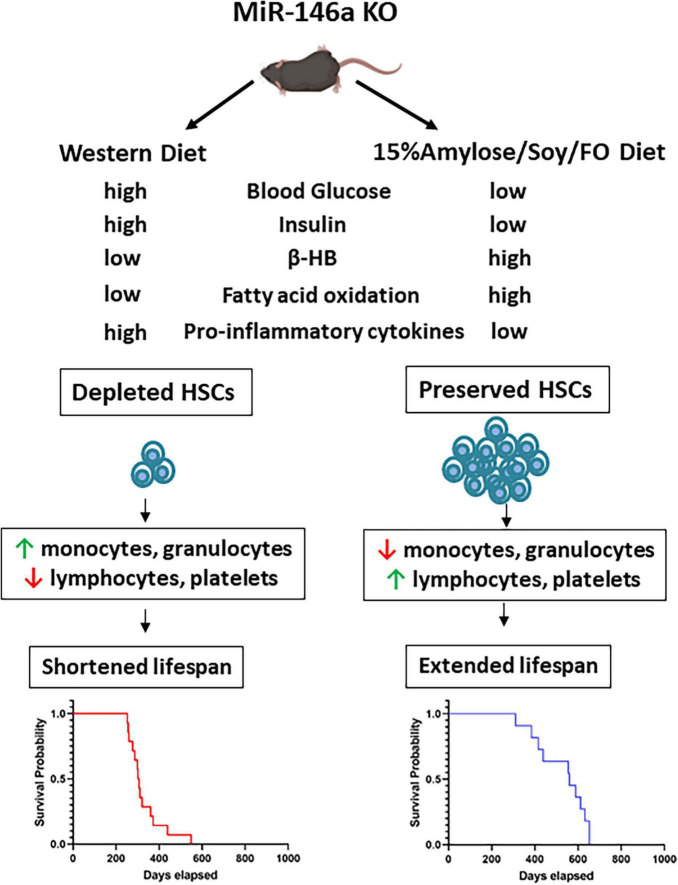
Proposed mechanisms by which a 15%Amylose/Soy/FO diet extends the lifespan of miR146a KO mice. In the absence of the anti-inflammatory microRNA, miR146a, monocytes and other myeloid cells express higher levels of pro-inflammatory cytokines. These cytokines induce miR146a KO HSCs to proliferate and differentiate, especially down the myeloid lineage. Over time, the HSCs are depleted and the mice die due to myelo-malignancies. Consumption of a 15%Amylose/Soy/FO reduces the levels of pro-inflammatory cytokines as well as blood glucose, and insulin and promotes FAO. Together, this preserves the pool of HSCs and ameliorates the aberrant blood cell profile associated with miR146a KO. This delays meylo-malignancy-induced death in these mice.

Based on our results we propose the model shown in Figure 7 in which the absence of the anti-inflammatory microRNA, miR-146a leads to increased pro-inflammatory cytokines, primarily from monocytes and other myeloid cells, when these mice are on a Western diet. These cytokines directly stimulate HSC proliferation and differentiation (36). Compounding this, the absence of miR-146a in HSCs makes them more sensitive to IL-6-induced cycling (10). Together, it is likely that this increases the short-term output of mature effector leukocytes but eventually leads to HSC exhaustion that likely lead to myeloid malignancy typically observed in MDS (10, 37, 38). In addition to modulating inflammation, this low CHO diet appears to push the mice to rely on FAO to obtain energy rather than glucose. This is known to promote asymmetric divisions of HSCs and thus helps to prevent their exhaustion (34, 35). It is thus likely that the reason the 15%Amylose/Soy/FO diet dramatically extends the lifespan of miR-146a mice is multi-factorial in nature.

One of the limitations of our study is that the Western and 15%Amylose/Soy/FO diets are very different in composition. Thus, we cannot pinpoint the specific dietary component(s) that contribute to the outcomes in this study. For example, the 15%Amylose/Soy/FO diet contains fish oil and soy protein, both of which we reported previously to be superior to other fats and casein, respectively, in preventing NNK-induced lung tumor formation in A/J mice (2). Of note, however, we have preliminary evidence that the superiority of SPI over casein may not be due to the protein components themselves but more likely the isoflavones and/or saponins in the SPI. Other components that differ between the two diets and may contribute to inflammation levels include calcium and insoluble fiber, which are lower in the Western diet (see Supplementary Tables 1–4). Future experiments in our laboratory include efforts to identify the specific dietary components in the 15%Amylose/Soy/FO that are most impactful in extending the lifespan of these miR-146a KO mice, as well as to determine if a reduction in chronic inflammation or a shift in metabolism is the dominant mechanism preserving the HSCs in these mice.

In summary, we found that a simple diet change from a Western to a low CHO diet containing soy protein and FO was effective in extending the lifespan of miR-146 KO mice. This was likely attributable, at least in part, to a reduction in chronic inflammation and a promotion of FAO which, in turn, potentially protected HSCs from stem cell exhaustion and myelo-malignancy-induced death.

## Data availability statement

The original contributions presented in this study are included in the article/Supplementary material, further inquiries can be directed to the corresponding author.

## Ethics statement

This animal study was reviewed and approved by the University of British Columbia Animal Care Committee (A18-0138).

## Author contributions

GK and IE conceived the project. IE, JG, and GK designed the experiments. IE, SK, MY, and JW performed the experiments. IE, MY, SK, JG, and GK analyzed the data and interpreted the results. JG and AK provided the technical expertise, and mice and intellectual contributions. IE, JG, AK, and GK wrote the manuscript. All authors contributed to the article and approved the submitted version.
