# Supplementary material for: A low carbohydrate diet high in fish oil and soy protein delays inflammation, hematopoietic stem cell depletion, and mortality in miR-146a knock-out mice
Source: Front Nutr. 2022 Nov 24;9:1017347. doi: 10.3389/fnut.2022.1017347 (PMC9729559; doi:10.3389/fnut.2022.1017347)
Supplement: Supplementary file 1 [file Presentation_1.pdf]

Supplementary Material

1 Supplementary Figures

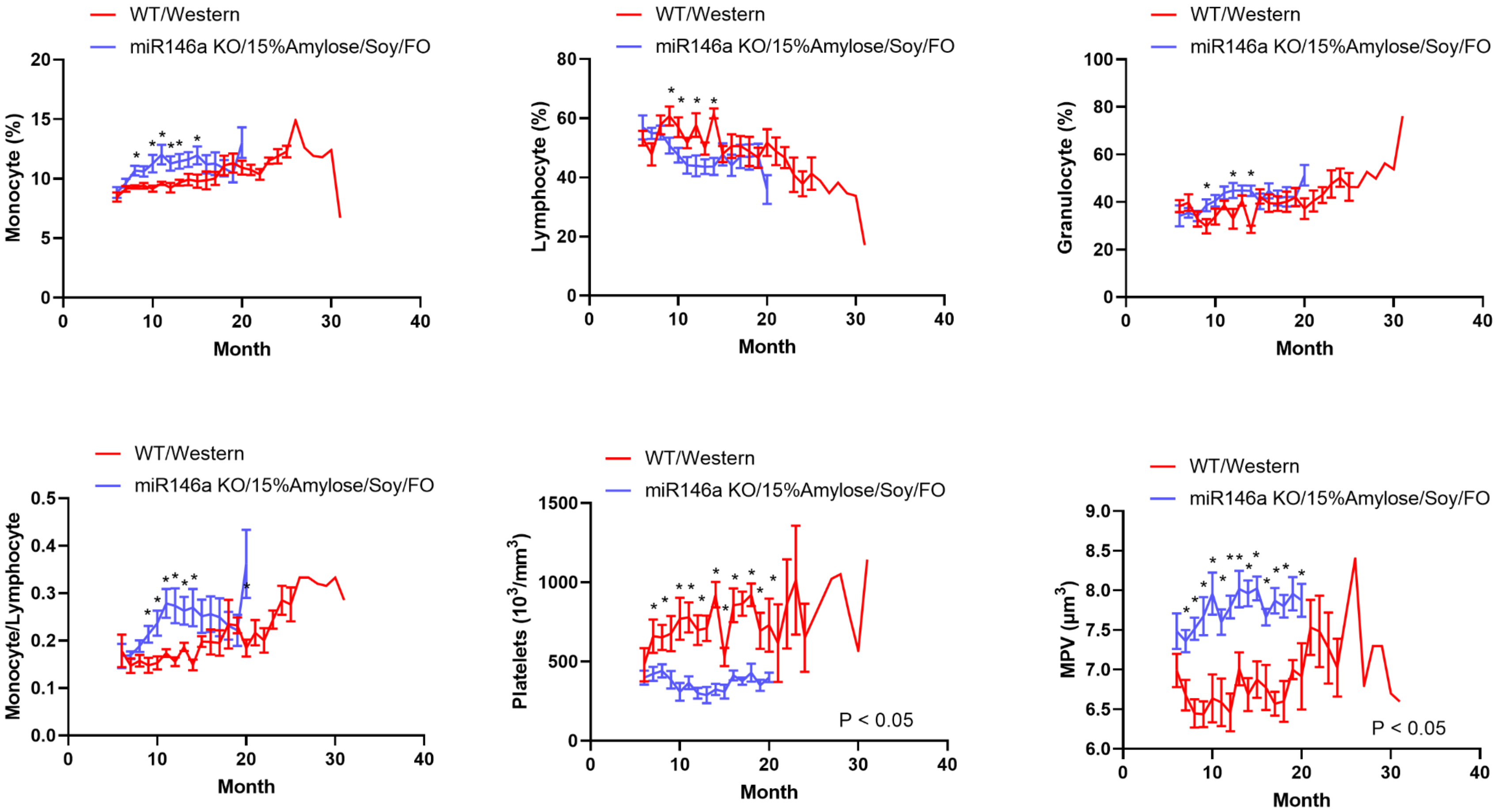

Supplementary Figure 1. Consumption of a 15%Amylose/Soy/FO diet brings the blood profiles of miR146a KO mice closer to that observed in WT mice. WT mice were fed a Western diet. * indicates significant differences (P<0.05).

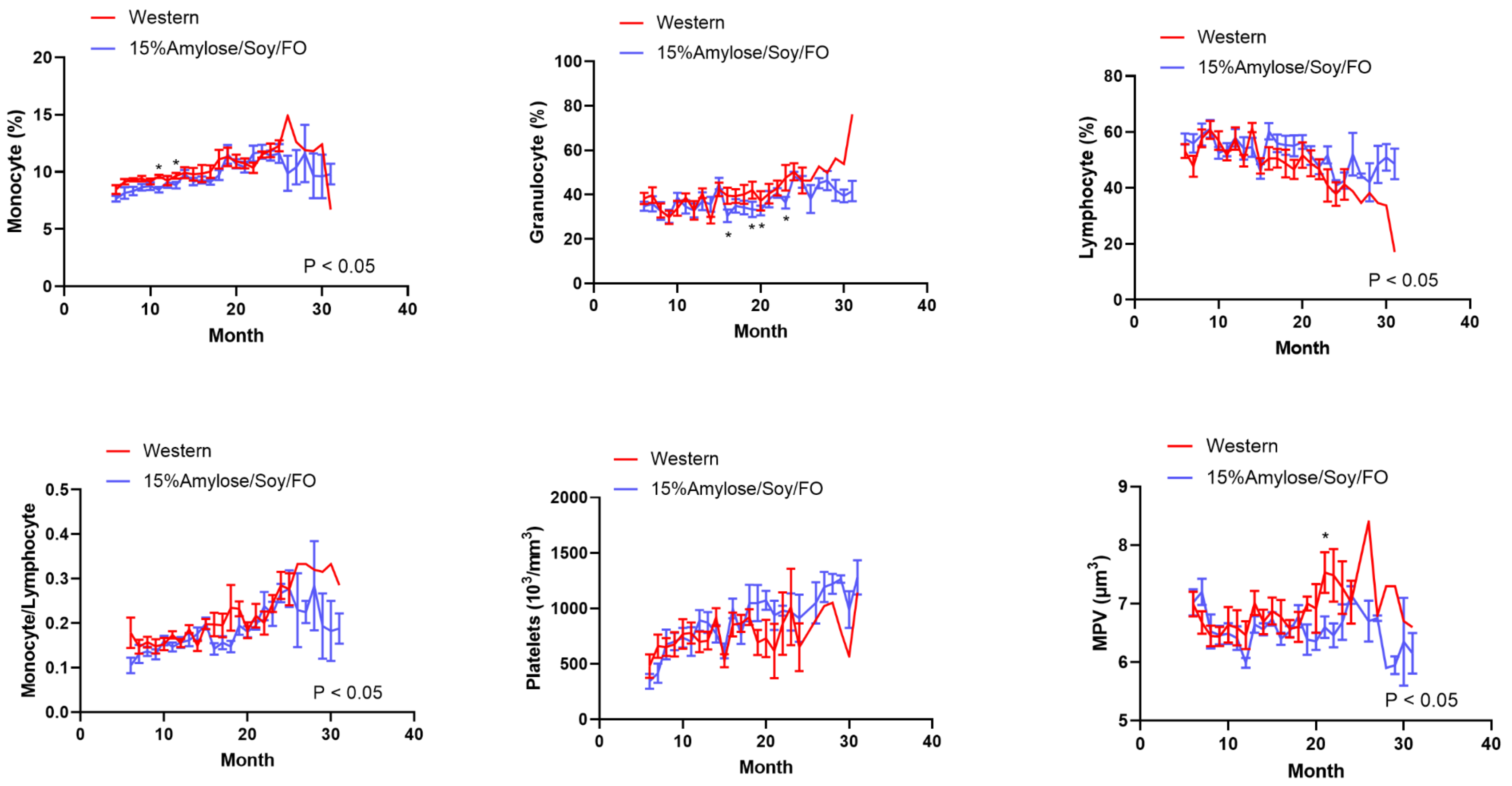

Supplementary Figure 2. There are only minor differences in blood cell profiles when WT mice are on a Western versus a 15%Amylose/Soy/FO diet. Shown is a comparison of the lymphocytes, monocytes, granulocytes and platelets over time in WT mice on a Western (n = 10) versus a 15%Amylose/Soy/FO (n = 10) diet. The data are shown as the means ± SEM. * indicates significant differences (P <0.05).

miR-146a KO - Humane Endpoint

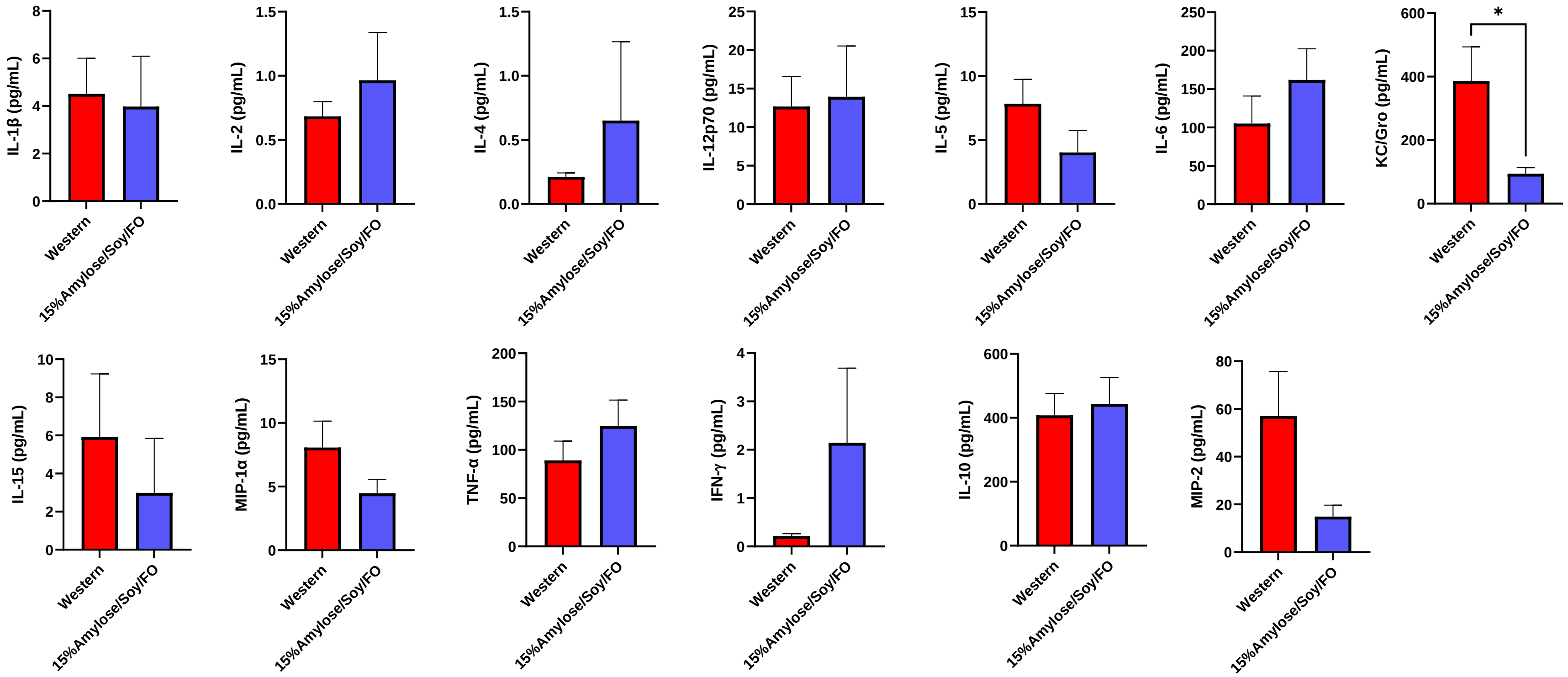

Suppl Fig 3. There are only minor differences in inflammatory cytokines when miR-146a KO mice on a Western versus a 15%Amylose/Soy/FO diet are compared at their humane endpoints. Luminex analysis of IL-1β, IL-2, IL-4, IL-5, IL-6, KC/Gro, TNFα, IFNγ and IL-10 plasma levels from miR-146a KO mice on a Western versus a 15%Amylose/Soy/FO diet at their human endpoints. The data are shown as the mean ±SEM of 15 miR146a KO mice on a Western and 15 miR146a KO mice on a 15%Amylose/Soy/FO diet. * indicates significant differences ($P < 0.05$).
