# Supplementary material for: A low carbohydrate diet high in fish oil and soy protein delays inflammation, hematopoietic stem cell depletion, and mortality in miR-146a knock-out mice
Source: Front Nutr. 2022 Nov 24;9:1017347. doi: 10.3389/fnut.2022.1017347 (PMC9729559; doi:10.3389/fnut.2022.1017347)
Supplement: Supplementary file 2 [file Table_1.DOCX]

| **Supplementary Table 1. Amino acid profile in diets expressed as g/kg of diet** | | | |
| --- | --- | --- | --- |
|  |  | **Western** | **15% Amylose/Soy/FO** |
|  |  |  |  |
|  | |  |  |
|  | Lysine | 13.3 | 21.8 |
|  | Methionine 4.4 | | 4.4 |
|  | Cystine | 3.3 | 7.2 |
|  | Arginine | 6.3 | 26.1 |
|  | Phenylalanine | 8.4 | 17.8 |
|  | Tyrosine | 8.7 | 13.1 |
|  | Histidine | 4.8 | 9.1 |
|  | Isoleucine | 9.5 | 17.0 |
|  | Leucine | 15.2 | 28.1 |
|  | Threonine | 7.2 | 13.1 |
|  | Tryptophan | 1.9 | 4.8 |
|  | Valine | 11.4 | 17.4 |
|  | Aspartic Acid | 11.4 | 40.0 |
|  | Glutamic Acid | 34.6 | 65.7 |
|  | Alanine | 4.9 | 14.7 |
|  | Glycine | 3.0 | 14.7 |
|  | Proline | 17.1 | 17.4 |
|  | Serine | 9.5 | 17.8 |
|  |  |  |  |
|  |  |  |  |
