# Supplementary material for: A low carbohydrate diet high in fish oil and soy protein delays inflammation, hematopoietic stem cell depletion, and mortality in miR-146a knock-out mice
Source: Front Nutr. 2022 Nov 24;9:1017347. doi: 10.3389/fnut.2022.1017347 (PMC9729559; doi:10.3389/fnut.2022.1017347)
Supplement: Supplementary file 3 [file Table_2.DOCX]

| **Supplementary Table 2. Mineral profile in diets expressed as g/kg of diet** | | | |
| --- | --- | --- | --- |
|  |  | **Western** | **15% Amylose/Soy/FO** |
|  |  |  |  |
|  | |  |  |
|  | Calcium | 2.0 | 2.8 |
|  | Phosphorus 2.7 | | 4.7 |
|  | Potassium | 5.2 | 5.8 |
|  | Sodium | 7.0 | 11.0 |
|  | Chlorine | 10.8 | 10.8 |
|  | Magnesium | 0.585 | 0.822 |
|  | Copper | 2.6 | 7.8 |
|  | Iron | 31.3 | 82.8 |
|  | Zinc | 24.6 | 44.8 |
|  | Manganese | 10.5 | 17.3 |
|  | Iodine | 0.21 | 0.21 |
|  | Selenium | 0.21 | 0.21 |
|  | Molybdenum | 0.15 | 0.15 |
|  | Chromium | 1.00 | 1.00 |
|  |  |  |  |
