# Supplementary material for: A low carbohydrate diet high in fish oil and soy protein delays inflammation, hematopoietic stem cell depletion, and mortality in miR-146a knock-out mice
Source: Front Nutr. 2022 Nov 24;9:1017347. doi: 10.3389/fnut.2022.1017347 (PMC9729559; doi:10.3389/fnut.2022.1017347)
Supplement: Supplementary file 4 [file Table_3.DOCX]

| **Supplementary Table 3. Fatty acid profile in diets expressed as g/kg of diet** | | | |
| --- | --- | --- | --- |
|  |  | **Western** | **15% Amylose/ Soy/FO** |
|  |  |  |  |
| **Total Fat** | |  |  |
|  | Saturated Fat | 59.7 | 62.4 |
|  | Monounsaturated Fat 68.0 | | 60.6 |
|  | Polyunsaturated Fat | 37.0 | 88.1 |
|  | 4:0 | 1.4 | 0.5 |
|  | 6:0 | 0.8 | 0.3 |
|  | 8:0 | 0.4 | 0.1 |
|  | 10:0 | 0.7 | 0.2 |
|  | 12:0 | 1.1 | 0.4 |
|  | 14:0 | 5.5 | 13.1 |
|  | 14:1 | 0.3 | 0.1 |
|  | 15:0 | 0.9 | 1.5 |
|  | 16:0 | 32.2 | 34.7 |
|  | 16:1 | 2.7 | 16.5 |
|  | 17:0 | 0.6 | 1.3 |
|  | 17:1 | 0.2 | 0.1 |
|  | 18:0 | 15.7 | 10.1 |
|  | 18:1 | 64.3 | 41.6 |
|  | 18:2 | 33.3 | 30.3 |
|  | 18:3 | 3.5 | 6.1 |
|  | 18:4 | 0.0 | 4.7 |
|  | 20:0 | 0.3 | 0.3 |
|  | 20:1 | 0.4 | 1.9 |
|  | 20:2 | 0.0 | 0.4 |
|  | 20:3 | 0.0 | 0.5 |
|  | 20:4 | 0.2 | 3.2 |
|  | 20:5 (EPA) | 0.0 | 21.5 |
|  | 21:5 | 0.0 | 1.1 |
|  | 22:1 | 0.0 | 0.4 |
|  | 22:4 | 0.0 | 5.2 |
|  | 22:5 (DPA) | 0.0 | 14.5 |
|  | 22:6 (DHA) | 0.0 | 0.7 |
|  |  |  |  |
