# Supplementary material for: A low carbohydrate diet high in fish oil and soy protein delays inflammation, hematopoietic stem cell depletion, and mortality in miR-146a knock-out mice
Source: Front Nutr. 2022 Nov 24;9:1017347. doi: 10.3389/fnut.2022.1017347 (PMC9729559; doi:10.3389/fnut.2022.1017347)
Supplement: Supplementary file 5 [file Table_4.DOCX]

|  |  | **15% Amylose/Soy/FO** |
| --- | --- | --- |
|  |  |  |
| **Total Isoflavones** | |  |
|  | Daidzin (aglycone units) | 520 |
|  | Daidzein (aglycone units) | 134 |
|  | Genistin (aglycone units) | 1120 |
|  | Genistein (aglycone units) | 132 |
|  | Glycitin (aglycone units) | 96 |
|  | Glycitein (aglycone units) | 16 |
|  |  |  |

**Supplementary Table 4. Isoflavone profile in diet expressed as mg/kg**
